# Organic Matter Degradation Drives Benthic Cyanobacterial Mat Abundance on Caribbean Coral Reefs

**DOI:** 10.1371/journal.pone.0125445

**Published:** 2015-05-05

**Authors:** Hannah J. Brocke, Lubos Polerecky, Dirk de Beer, Miriam Weber, Joachim Claudet, Maggy M. Nugues

**Affiliations:** 1 Department of Biogeochemistry, Max Planck Institute for Marine Microbiology (MPI Bremen), Bremen, Germany; 2 Department of Ecology, Leibniz Center for Tropical Marine Ecology (ZMT), Bremen, Germany; 3 Laboratoire d’Excellence Corail, CRIOBE—USR 3278, EPHE-CNRS-UPVD, Perpignan, France; 4 Geochemistry, Faculty of Geosciences, Utrecht University, Utrecht, The Netherlands; 5 HYDRA Institute for Marine Sciences, Elba Field Station, Campo nell'Elba, Italy; 6 Caribbean Research and Management of Biodiversity (CARMABI) Foundation, Willemstad, Curaçao, Netherlands Antilles; Biodiversity Research Center, Academia Sinica, TAIWAN

## Abstract

Benthic cyanobacterial mats (BCMs) are impacting coral reefs worldwide. However, the factors and mechanisms driving their proliferation are unclear. We conducted a multi-year survey around the Caribbean island of Curaçao, which revealed highest BCM abundance on sheltered reefs close to urbanised areas. Reefs with high BCM abundance were also characterised by high benthic cover of macroalgae and low cover of corals. Nutrient concentrations in the water-column were consistently low, but markedly increased just above substrata (both sandy and hard) covered with BCMs. This was true for sites with both high and low BCM coverage, suggesting that BCM growth is stimulated by a localised, substrate-linked release of nutrients from the microbial degradation of organic matter. This hypothesis was supported by a higher organic content in sediments on reefs with high BCM coverage, and by an *in situ* experiment which showed that BCMs grew within days on sediments enriched with organic matter (*Spirulina*). We propose that nutrient runoff from urbanised areas stimulates phototrophic blooms and enhances organic matter concentrations on the reef. This organic matter is transported by currents and settles on the seabed at sites with low hydrodynamics. Subsequently, nutrients released from the organic matter degradation fuel the growth of BCMs. Improved management of nutrients generated on land should lower organic loading of sediments and other benthos (e.g. turf and macroalgae) to reduce BCM proliferation on coral reefs.

## Introduction

Cyanobacteria are ubiquitous on coral reefs and play an important role in reef formation and nutrient cycling [1]. However, on declining reefs, they can form dense and widespread benthic cyanobacterial mats (BCMs), with negative consequences for reef health [1,2]. Since the early 1990s, BCMs have become increasingly prominent on many reefs worldwide, including Australia [3], California [4], Florida [5], Guam [6], Hawaii [7], La Reunion [8], New Caledonia [9], Taiwan [10] and Tuamotu Archipelago [11]. The mats reduce coral settlement and recruitment [12], alter coral-associated microbial communities [13], act as coral pathogens [14], and produce chemicals which have been linked to mass reef fish die-offs and deter grazing [15,16]. As many cyanobacteria are able to fix nitrogen (N) [17], their proliferation could also increase fixed nitrogen in the system, which may enhance the growth of coral competitors, such as macroalgae [18].

The ability of BCMs to tolerate environmental conditions associated with anthropogenic impacts and global climate change has been suggested to explain their increasing abundance on degraded reefs [2,19]. However, the links between potential anthropogenic and climate drivers and the proliferation of BCMs on coral reefs are not supported by a mechanistic explanation. Water column measurements in coral reefs with naturally growing BCMs have not shown elevated inorganic nutrient concentrations [20,21], including in reefs considered under eutrophic exposure [22], probably because these inorganic nutrients are rapidly converted into biomass. Since many BCMs are diazotrophs [23], their growth is unlikely to be limited by N. Manipulative studies on BCMs present on coral reefs are few, but suggest that they may be limited by phosphorus (P) [6,24], chelated iron (Fe) [25] or mixed N and P [26–28]. However, the sources and transport routes of nutrients which enhance BCMs are unclear.

In Moreton Bay, Australia, a bloom of the benthic cyanobacterium *Lyngbya majuscula*, which also proliferates on coral reefs [5,6], was preceded by a pulse of rainfall and initiated by a period of high incident light, elevated temperature and calm weather [29]. Soil extracts rich in P, Fe and organic carbon enhanced the productivity of *L*. *majuscula* in bioassays, suggesting land runoff as a key driver of the bloom [3]. Increased dissolved organics in the water column may facilitate the transport of bio-available Fe and P to *L*. *majuscula* via the formation of Fe-organic complexes [30,31]. Recently, Fe released from corroding shipwrecks was suggested to stimulate algal/cyanobacterial assemblages in central Pacific reefs through a similar mechanism [32]. Given that BCMs bloom on the benthos, they could also acquire benthic nutrients released from macrofaunal excretions, groundwater seeps and remineralised organic matter (OM) [18,33]. High effluxes of both P and N have been measured in benthic cores containing *L*. *majuscula*, seagrasses and sediments, highlighting the potential of sediments to act as local supply of nutrients for uptake [29].

This study aimed to improve our understanding of the factors and mechanisms driving the proliferation of BCMs on coral reefs in Curaçao, Southern Caribbean. Over the last three decades, many reefs on the island have exhibited signs of degradation and increasing BCM dominance [34]. We hypothesised that OM degradation acts as a mechanism of nutrient supply and growth impulse for benthic cyanobacterial mats in nutrient-poor coral reefs and that coastal urbanisation and hydrodynamics combine to mediate the accumulation of particulate organic matter on the seafloor, which subsequently stimulates the growth of the mats. This study is based on (i) large-scale surveys of BCM, coral and algal abundance around the island, (ii) local surveys of potential environmental drivers, including inorganic nutrient concentrations, temperature, water movement and OM content in sediments, and (iii) an *in situ* organic enrichment experiment of the sediments. In addition, we used microsensors to estimate microbial activity and degradation of OM across BCM patches. From these results, we deduced the possible sources and transport mode of nutrients stimulating BCM growth.

## Material and Methods

### Study area

**Fig 1:**
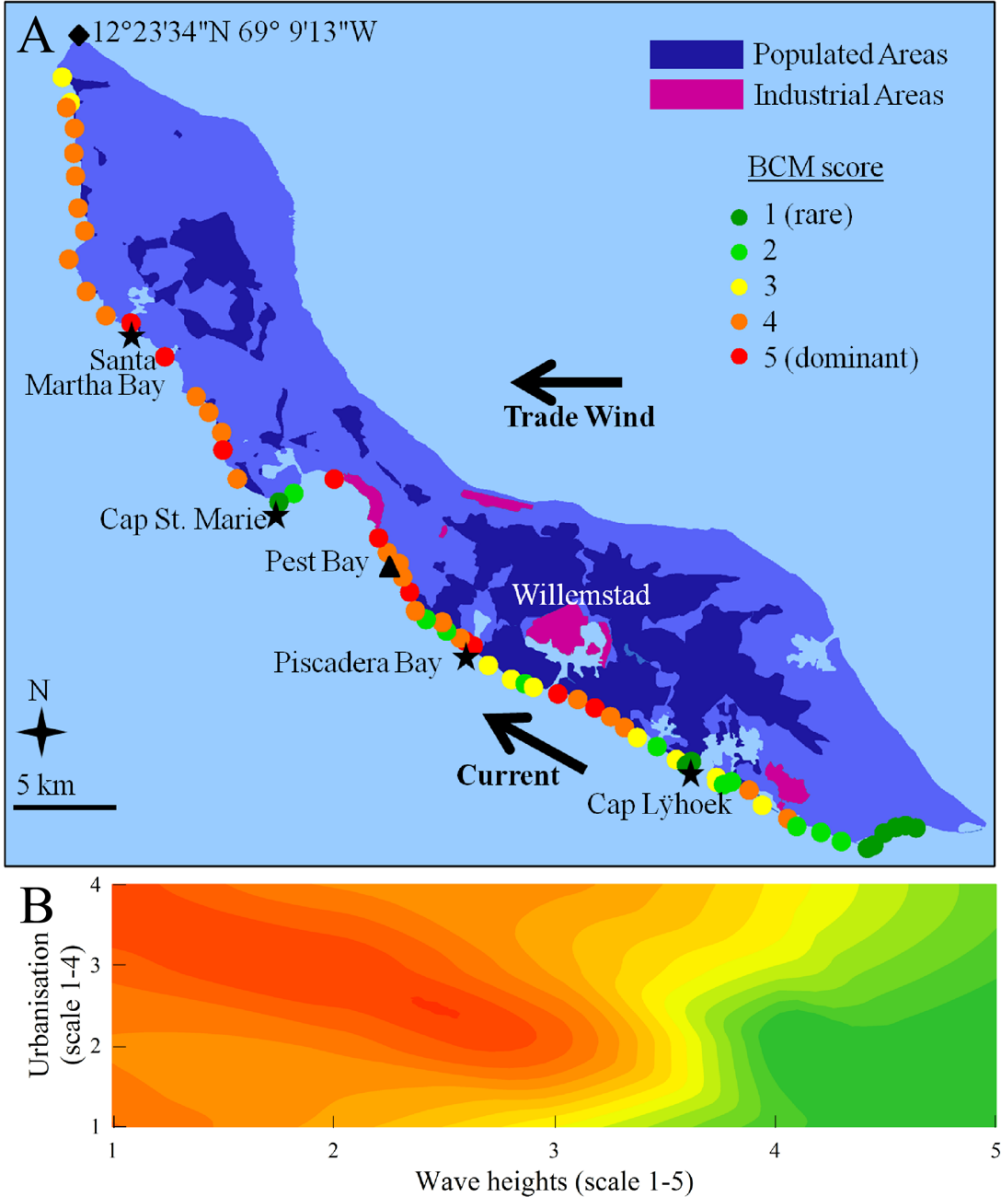
BCM abundance along the coast of Curaçao and relationship with wave height and urbanisation. (A) BCM abundance score (scale 1 to 5) averaged across the 4 surveys along the south coast of Curaçao. Populated and industrial areas are shown in dark blue and pink, respectively. Trade wind and water current are indicated by arrows. Stars locate the sites of low and high abundance of BCMs used as examples in the Results. The triangle locates the site of the *in situ* organic enrichment experiment. The diamond shows a site with GPS coordinates to locate the study area. (B) Contour plot showing the relationships between BCM abundance, wave height and urbanisation. Colours represent BCM score as shown in (A). Note that populated areas and BCM abundance are not obviously related in the large-scale map (A), especially in the West part of the island, but populated areas were often present along a narrow (ca. 500 m wide) strip of coast, which cannot be visualised in the map. This small-scale pattern was taken into account to score urbanisation levels used in the contour plot (B) (see Materials and Methods for details).

The research was conducted on the south leeward coast of the southern Caribbean island of Curaçao (12°10’N, 68°58’W, ca. 60 x 11km, Fig 1A), where well-developed fringing reefs border the landward shore [35]. The island is exposed to all-year-around trade winds running from east to west [36]. In 2012, 150,563 inhabitants lived on the island (Central Bureau of Statistics, Curaçao). Curaçao does not have large scale agriculture, but heavy oil industry and mining activity are present. In the east and central parts of the island, waste water treatments are installed, but receptive basins have leaks and overflow regularly, which makes household wastewater runoff a significant source of nutrients into the ocean [37]. In the west part of the island, most houses have sewage cesspits that leak slowly into the groundwater. The island is surrounded by a belt of Quaternary and Neogene limestone [38], which is a porous material that allows fast groundwater transport. Permission to conduct our studies was provided by the Ministry of Health, Environment and Nature (GMN) of the government of Curaçao through their permit (#48584) to the Caribbean Marine Biological Institute (CARMABI) at Willemstad.

### Large-scale survey

To provide an island-wide view of BCM abundance and potential drivers, we conducted semi-quantitative multi-season surveys along the entire south-west coast of Curaçao and linked our observations to coastal urbanisation, wave action and seasonality. These large-scale surveys were coupled with local surveys of selected environmental parameters at haphazardly chosen sites with high and low BCM abundance.

The semi-quantitative surveys of BCM abundance were conducted at 64 sites over four periods: twice during the warmer and rainier season (Sept 2010 and November 2011) and twice during the colder and drier season (May 2011 and June 2012). At each site and period, the observer first dove to 20 m depth and then slowly swam upwards to 5 m depth at a distance of ca. 2 m from the reef while watching attentively the seabed (observation time: ~3 min per site). The abundance of BCMs was ranked on a scale of 1 to 5 where 1 corresponds to a reef where cyanobacteria are rare (i.e. a small patch could occasionally be seen) and 5 represents a reef where most sand and hard bottom surfaces are covered by BCMs. During the last survey (June 2012), the abundance of corals and macroalgae (defined as all algae extending more than 1 cm above the reef substratum) was similarly ranked at each site to investigate their spatial relationships with BCMs. The lead observer (HJB) led all surveys and the ranking was cross-validated between the different divers at the beginning of each survey period. In addition, representative mats from two different depths (5 and 15 m) were collected at several study sites and analysed microscopically to identify dominant species.

Each site was assigned two individual scores: one score for the level of urbanisation of the adjacent shore and one for the wave height. For urbanisation, each site was plotted in Google Earth Pro and urbanisation level was defined as follows: 1, absence of urbanisation (i.e. absence of houses, industry, dumping areas) in a 500 m radius; 2, presence of urbanisation in a 500 m radius; 3, presence of a drainage outlet with an urbanised watershed in the 500 m radius; and 4, both 2 and 3. The 500 m radius prevented overlap between sites. For wave heights, the semi-quantitative estimates of van Duyl [35] were used, where 1 represents low wave energy environments (waves 0–30 cm high) and 5, high wave energy environments (waves 1.5–2 m high).

### Local surveys of environmental parameters

Based on the results of the island-scale surveys, four sites with high (rank 4–5) and four sites with low (rank 1) BCM abundance were randomly selected in subsequent, medium-scale surveys of selected environmental parameters. If the site characteristic (i.e., high vs. low mat abundance) changed during the multi-season survey, another site that fulfilled the required classification criterion was selected. The parameters monitored during these medium-scale surveys included temperature, water movement, nutrients (NO_x_, PO_4_^3-^), particulate organic matter in the water column and OM content in sediments. Temperature was recorded in 30 min intervals from September 2010 to June 2012 at a water depth of 10 m using temperature loggers (Hobo Pendant, Onset). Water movement was estimated in September 2010 and May 2011 at a water depth of 10 m based on the dissolution of clodcards (i.e. plaster of paris blocks; [39]). These data were used as an additional support for the above wave height data.

#### Nutrients

**Fig 2:**
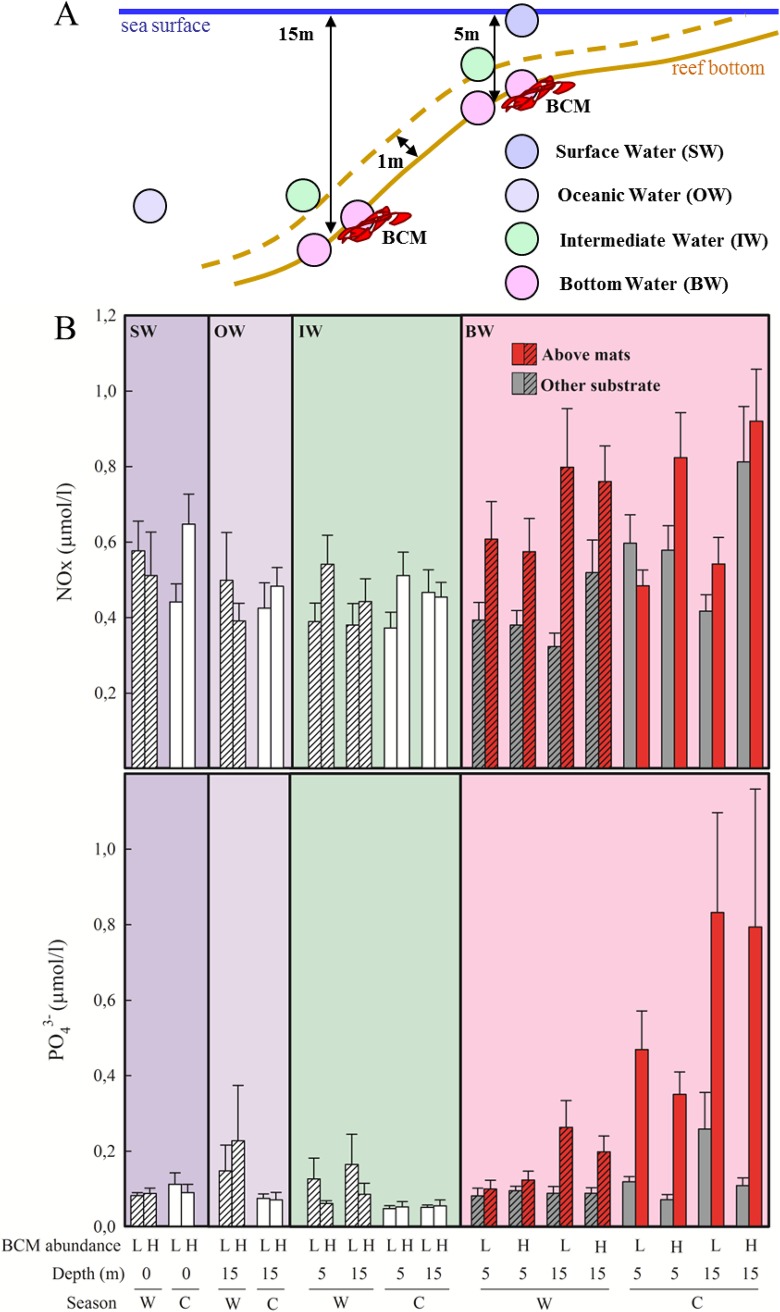
Nutrient concentrations in the water column. (A) Overview of different nutrient sampling locations at each site. (B) NO_x_ and PO_4_^3-^ concentrations (mean ± SEM, n = 3–4 temporal replicates) as a function of season, ie. (W) warm/rainy (hatched) and (C) cold/dry (plain), depth, BCM abundance, ie. low (L) vs. high (H) abundance sites, and substrate type (as applicable) for surface, open ocean, intermediate and bottom waters.

Nutrients were analysed in water samples collected 3–4 times during a warmer and rainier season (October-November 2010) and 3–4 times during a colder and drier season (April-May 2011). To identify possible differences that could be relevant at the medium scale, samples were collected at each site in 8 locations (Fig 2A): (1) above the reef slope just below the water surface (surface water); (2) 15 m away from the reef slope at a water depth of 15 m (open ocean water); above the reef slope, 1 m above the seabed at water depths of 5 m (3) and 15 m (4) (intermediate water); directly above BCMs (within 1 cm of the seabed) at water depths of 5 m (5) and 15 m (6) (bottom water), and directly above BCM-free substrate at water depths of 5 m (7) and 15 m (8) (control bottom water). Control bottom water samples at 5 and 15 m depths were taken above sand and hard substrate, respectively. Since mats differed in substrate preference and species composition across depths, the effects of depth, substrate and mat type were unavoidably confounded. Immediately upon collection, samples were filtered with 0.22 μm pore-size syringe filters (Minisart NML Syringe Filters 16534), transported on ice and in the dark to local laboratory (CARMABI), and stored and transported at -20 C° until analysis at MPI, Germany. PO_4_^3-^ was analysed using the molybdenum blue method [40] and NO_x_ (nitrate + nitrite) was analysed with a NO_x_ analyser (CLD 86; Eco- Physics).

#### Particulate organic matter content in water column

Water samples (2 l) were collected 4 times 1 m above the seabed at a depth of 10 m during a warmer and rainier season (October-November 2010) and filtered immediately after returning to the laboratory through a precombusted GFF filter. Each filter was separately packed in individual acid washed filter box and dried at 40°C. The filters were steamed with smoking hydrochloric acid for 24 h, dried again, packed in tin cups and analysed with a CNS elemental analyser.

#### Organic matter content in sediments

Sediment cores (6 cm^2^ area x 3 cm deep) were collected at a water depth of 7–8 m in April 2012 (n = 3 per site). For this parameter, 5 sites with high and 4 sites with low BCM abundance were sampled. In all sites, sampling was conducted on sand patches far away (> 5 m) from BCMs to minimize their potential influence. To study small-scale variations at the mat level, sediment cores were collected in the centre, at the edge, next to (ca. 10 cm away) and far away (> 5 m) from brown-coloured BCM patches (n = 6 patches per location) at one BCM dominant site (Pest Bay, 12°09’53.77” N 69°00’39.66”W, Fig 1A). Mats were removed by hand picking before sampling. Each sample was dried at 40°C, homogenised and analysed for organic carbon content with a Delta Plus mass spectrometer.

### In situ organic enrichment experiment

To test the hypothesis that a substrate-bound degradation of OM stimulates the growth of the BCMs, an *in situ* organic enrichment experiment was conducted in May 2012 at a depth of 6–9 m using brown-coloured mats in sandy areas in the patch reef at Pest Bay (Fig 1A). A total of 48 buckets (14 L) were installed with a minimum gap of 1 m between buckets (total experimental area was ca. 1000 m^2^). Half of them had the base removed and the other half were intact to test for the possible effects of seepage. Buckets were pushed approximately 20 cm into the sand, with the same sand level inside and outside the bucket. Each set of 24 buckets was subject to four treatments differing in the OM content of the sediment and the presence of an initial “seed” of brown-coloured BCM: (i) the experimental control, without addition of OM and BCM seed; (ii) the seeding treatment, with addition of BCM seed, but without OM enrichment; (iii) the OM enrichment treatment, with OM enrichment, but no addition of BCM seed; and (iv) the combined treatment with additions of both OM and BCM seed.

To realize these treatments, sediment from the upper 15–20 cm layer of all buckets was discarded and replaced by sediment that was collected at the same site, repeatedly flushed *in situ* with water and well mixed. The untreated sediment contained 2.5 ± 0.1 μg C_org_ mg^−1^ DW (0.27 ± 0.02 SEM % C_org_ of sediment DW) and was enriched with *Spirulina* tablets (1 per bucket, 18.2 ± 0.7 mg C_org_), resulting in an additional organic carbon content of +0.7 ± 0.2% C_org_ in the upper 1 cm of the sediment (n = 6 for all measurements). OM content was sampled and analysed with the same method mentioned above. To enrich the sediment with OM, each tablet was dissolved in filtered (0.22 μm pore-size) seawater in a 60 ml syringe and slowly injected into the first 0.5–1 cm surface sediment in a spiral movement from the centre of the bucket at day 0 and again at day 7. Initial BCM “seeds” were scooped out of a ca. 6 cm^2^ surface of BCMs growing at the experimental site and placed at the centre of the bucket 4 h after the first OM enrichment. To compare BCM growth, buckets were photographed daily for 11 days and once after 18 days. BCM cover was estimated using the free software Vidana which allowed BCMs to be delineated by eye in each bucket and their surface to be determined.

### In situ oxygen measurements and other sediment analyses

To document microbial activity and degradation of OM across BCM patches, vertical profiles of dissolved oxygen around the sediment-water interface were measured with a diver-operated microsensor system [41] at a depth of 6–9 m at Pest Bay (Fig 1A). Over 350 profiles were acquired during several 24 h cycles in the centre, at the edge and next to brown-coloured BCM patches. Analysis of the profiles was done using custom-made programs MPR-plotter and L@MP (www.microsen-wiki.net). To verify that differences in O_2_ concentration were solely due to variations in microbial activity within the mats and not due to differences in sediment structure, particle size distribution, porosity and permeability were investigated in carbonate sediments collected in sand patches with and without BCMs. Particle size distribution was determined in 9 replicate sediment cores (6 cm diameter x 18 cm high) at each location (i.e. centre, edge, next to BCM). Porosity was measured by weight loss after drying 7 replicate sediment cores (6 cm diameter x 10 cm high) sliced at 2.5 cm depth intervals. Permeability was measured in 7 replicate sediment cores (36 mm diameter) with the falling-head method [42].

### Statistical analysis

Most environmental data violated parametric assumptions, so we evaluated them using non-parametric univariate analyses. BCM abundance scores were analysed using PERMANOVA with season (colder vs. warmer) as a fixed factor, time periods nested within season as a random factor and scores of urbanisation and wave height as covariate. Spearman rank-order correlation analyses were conducted to examine relationships between BCM, corals and macroalgae. Clodcard dissolution was analysed by PERMANOVA with BCM site abundance (low vs. high) and time (colder vs. warmer) as crossed fixed factor and site nested within BCM site abundance as a random factor. Ocean, surface, intermediate and bottom water nutrients and particular organic matter in the water column were separately analysed using temporal replicates by PERMANOVA with, as appropriate for each variable, season, BCM site abundance, depth (5 vs. 15 m) and BCM presence (above BCM vs. above BCM-free substrate) as crossed fixed factors and site as a random factor nested within BCM abundance. Temperature time-series (n = 38000 per site) were averaged for each site and analysed with ANOVA with BCM site abundance as fixed factor. Particle size distribution was analysed by PERMANOVA with size fractions and BCM presence as fixed factors, porosity by ANOVA with sediment depth interval and BCM presence as fixed factors, and permeability by ANOVA with BCM presence as fixed factor only. Other parameters were tested as described in the figures. All PERMANOVA tests used Euclidian distances and 9999 permutations of raw data from residuals under a reduced model [43].

## Results

### Surveys of BCM abundance and environmental parameters

BCMs were rare at the southern-most part of the island and increased in abundance when moving in the north-west direction along the coast, particularly in the sheltered and densely populated areas close to Willemstad (Fig 1A). On the west part of the island away from Willemstad, BCMs were also abundant. This part did not exhibit large populated or industrialised areas, but second homes and tourism development was frequent along a narrow (ca. 500 m) strip of coast (i.e. not visible on the island map). Urbanisation and wave height interacted significantly to explain BCM abundance (S1 Table). Areas with high cyanobacterial mat abundance were associated with high urbanisation and low wave energy, while sites with lower coverage were related to high wave energy and low urbanisation (Fig 1B). Examples of high-BCM sites include sheltered bays such as Santa Martha Bay and Piscadera Bay; examples of low-BCM sites include eastward facing promontories capes of St. Marie and Lÿhoek (Fig 1A). The negative influence of wave height was further supported by clodcard measurements, which revealed higher water movement at sites with low BCM abundance than at sites with high BCM abundance (mean ± SEM: 64 ± 3% vs. 35 ± 2% weight loss; S2 Table). BCMs were more abundant during the warm/rainy season than during the cold/dry season (abundance score of 2.9 ± 0.1 vs. 2.6 ± 0.1; S1 Table). Seasonal fluctuations in water temperature were ~3°C (~26–29°C), while daily fluctuations were ~0.5°C (S1 Fig). Average temperatures did not differ between sites with high and low BCM abundance (27.94 ± 1.02°C vs. 27.91 ± 1.01°C; *F* = 0.15, *df* = 1, *P* = 0.71), indicating that temperature influenced seasonal, but not spatial, variation in BCM abundance. In June 2012, BCM abundance was negatively correlated with coral abundance (*r*_s_ = -0.691, *P* < 0.001) and positively correlated with macroalgal abundance (*r*_s_ = 0.555, *P* < 0.001).

Across depths, dominant mats differed in substrate preference and species composition. BCMs at 5 m depth occurred largely on sand and were mostly brown-coloured. Morphological microscopic identification indicated that they consisted primarily of *Oscillatoria bonnemaisonii* Crouan & Crouan ex Gomont, 1892 and secondarily of *Hydrocolium glutinosum* (Gomont ex Gomont) Anagnostidis & Komárek, 2001. BCMs at 15 m depth occurred predominantly on hard substrate and were red-coloured. They consisted of different species of the genus *Oscillatoria* which could not be identified to species level.

Local surveys of inorganic nutrients at eight locations within each site (Fig 2A) showed that nutrient concentrations in the open ocean and surface waters did not differ with season or between sites with low and high BCM abundance (Fig 2B; S3 Table). Intermediate water (i.e. 1 m above the reef substrate) had higher NO_x_ concentration at 5 m depth at sites with high BCM abundance than at sites with low BCM abundance. Most noticeable were the elevated PO_4_^3-^ bottom water concentrations above BCM compared with BCM-free substrate, especially during the cold/dry season. This trend occurred regardless of depth and BCM site abundance. NO_x_ bottom water concentrations above BCM and BCM-free substrates showed similar, but less marked, differences. NO_x_ concentrations above BCMs were higher than above BCM-free substrate at sites of high BCM abundance at 15 m depth, regardless of season. No such trend was found for PO_4_^3-^.

**Fig 3:**
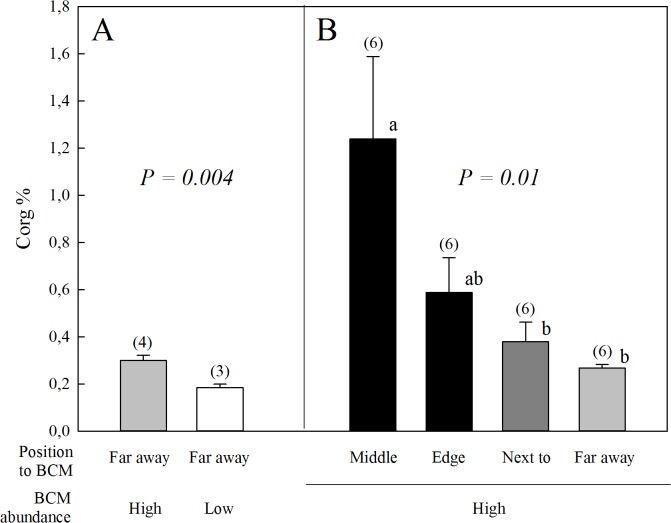
Organic matter content in sediments. Percent organic carbon (C_org_) (mean ± SEM) of (A) sediment cores collected far away (> 5 m) from any BCM patch at sites of low and high BCM abundance, and (B) sediment cores collected at Pestbay (site of high BCM abundance) in the middle, edge, next to (ca. 10cm away) and far away (> 5 m) from BCM patches. Analysed by ANOVA. Letters indicate homogeneous subgroups by posthoc Scheffe tests. Numbers of replicates are in parenthesis.

Average concentration of particulate organic matter in the water column did not differ between sites with low and high BCM abundance (28.91 ± 1.48 μg/l vs. 25.82 ± 1.22 μg/l; S4 Table). However, OM content in BCM-free sediments at sites with high BCM abundance was significantly higher than at sites with low BCM abundance (Fig 3A). It was also highest in the middle of BCMs and decreased with distance away from the mats, supporting an increased OM accumulation underneath the mats (Fig 3B).

### In situ organic enrichment experiment

**Fig 4:**
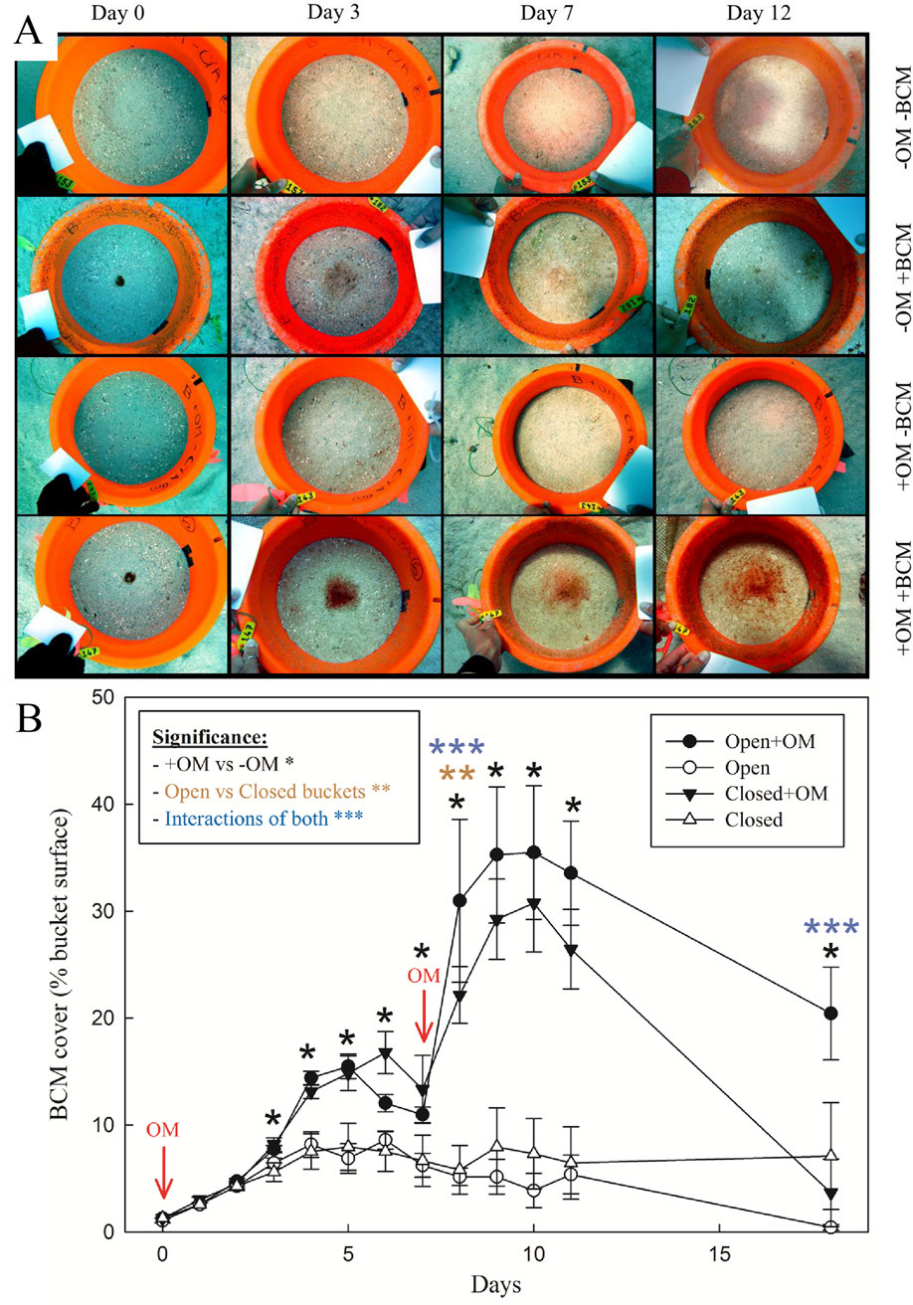
Response of BCMs to organic matter enrichment. (A) Representative photographs of the sediment surface for BCM seeded and non-seeded treatments with and without OM enrichment on days 0, 3, 7 and 12 (closed bucket). BCMs are visible as reddish brown coloration at the centre of the seeded buckets. (B) BCM coverage (mean ± SEM, n = 6) in open bottom and closed bottom buckets with initial BCM seed with and without OM enrichment over the duration of the experiment. Red arrows indicate when OM was added. Analysed by two-way ANOVA. Stars indicate significant differences.

Cyanobacterial growth occurred only on sediments that were seeded with a piece of BCM (Fig 4A; row 2 and 4). Starting from day 4 onwards, the cover of BCM seeded on OM-enriched sediments was significantly higher than on non-enriched sediments (Fig 4B). Differences between the buckets with open and closed bottoms were mostly not significant, although at certain time-points the open bottom buckets with the OM-enriched sediment had a significantly larger BCM coverage than the closed bottom buckets (e.g., day 9 or 18). BCM cover peaked on days 5 and 6 in the OM enriched open and closed bottom treatments after the first OM addition. Both also showed a large increase in cover after the second OM addition on day 7, with a peak in abundance on days 9 and 10 and a decline afterwards. The open bottom buckets showed a slower decline in BCM cover than the closed bottom buckets.

### In situ oxygen measurements and other sediment analyses

**Fig 5:**
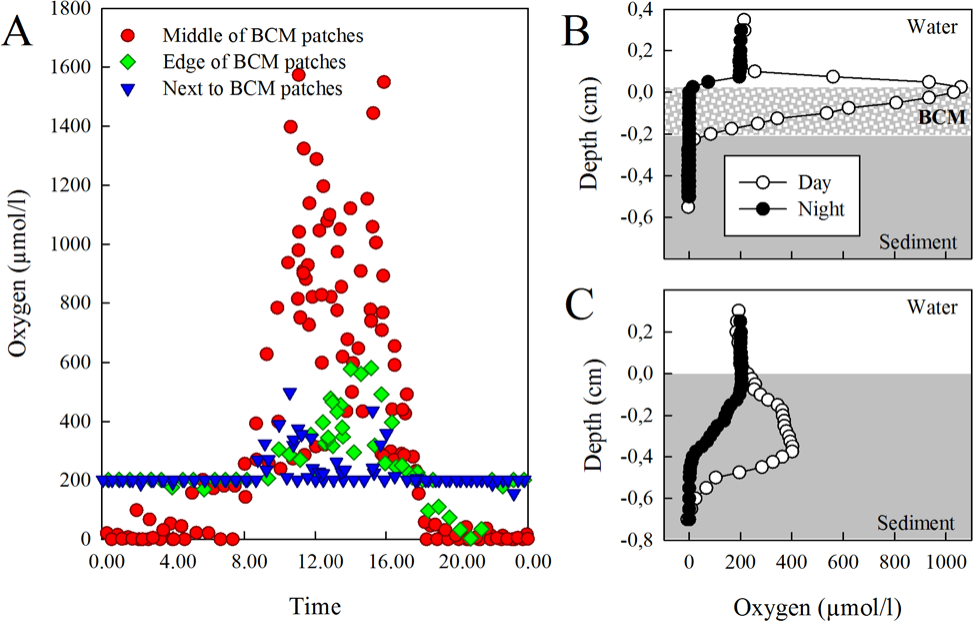
Oxygen concentrations in sediments across BCM patches. (A) O_2_ concentration across BCM patches over an entire diel cycle. During photosynthesis, O_2_ peak concentrations per profile are plotted; otherwise surface concentrations are given (n = 350 profiles). (B and C) Examples of *in situ* O_2_ profile across the water-sediment interface during day and night with BCM (B) and without BCM (C).

During daytime, maximum surface O_2_ concentrations in the middle of the natural BCM patches were 2–8 fold higher than at the edge and next to BCMs (Fig 5A), indicating high photosynthetic productivity in the mats. During night time, the mats were fully anoxic, whereas O_2_ penetrated 3–4 mm into the sediments in the absence of BCMs (compare Fig 5B and 5C). No differences in grain sizes (regardless of size fraction), porosity and permeability were found (PERMANOVA/ANOVA, all factors and interactions, *P* > 0.05). Permeability averaged 2.4 ± 0.2 x10^-10^ m^2^, indicating very permeable sediments.

## Discussion

BCM growth on coral reefs is typically limited by nutrients [6,24–27]. In our study, BCMs were congruent with high PO_4_^3-^ and NO_x_ in their close vicinity (millimetre-scale) in an otherwise oligotrophic water column (NO_x_ ~0.5 μM; PO_4_^3-^ ~0.1 μM), regardless of depth and BCM dominance on an island scale. Such locally enhanced nutrient concentrations could originate from the underlying benthic surface via direct groundwater seepage. In Australia, *L*. *majuscula* showed increased growth by exposure to groundwater [44]. However, in our case, several observations are inconsistent with this mechanism of nutrient supply. First, whereas seepage will be less in hard substratum which is much less permeable than sand, BCMs were prominent over hard substratum at 15 m depth where they were also associated with locally enhanced nutrient concentrations. Second, our nutrient sampling periods were in September 2010 and May 2011 which experienced 195 mm/month and 53mm/month of rain, respectively (Meteorological Dept, Curaçao). Yet, higher concentrations of nutrients were found in the dryer sampling period when any possible groundwater seepage would have been reduced. Third, differences between the buckets with open and closed bottoms in the organic enrichment experiment were mostly not significant. Finally, while NO_x_ benefits from a high mobility in sediments, PO_4_^3-^ in groundwater tends to get absorbed by calcium present in limestone and should thus be low in groundwater outflow [45], which contrasts with our data.

**Fig 6:**
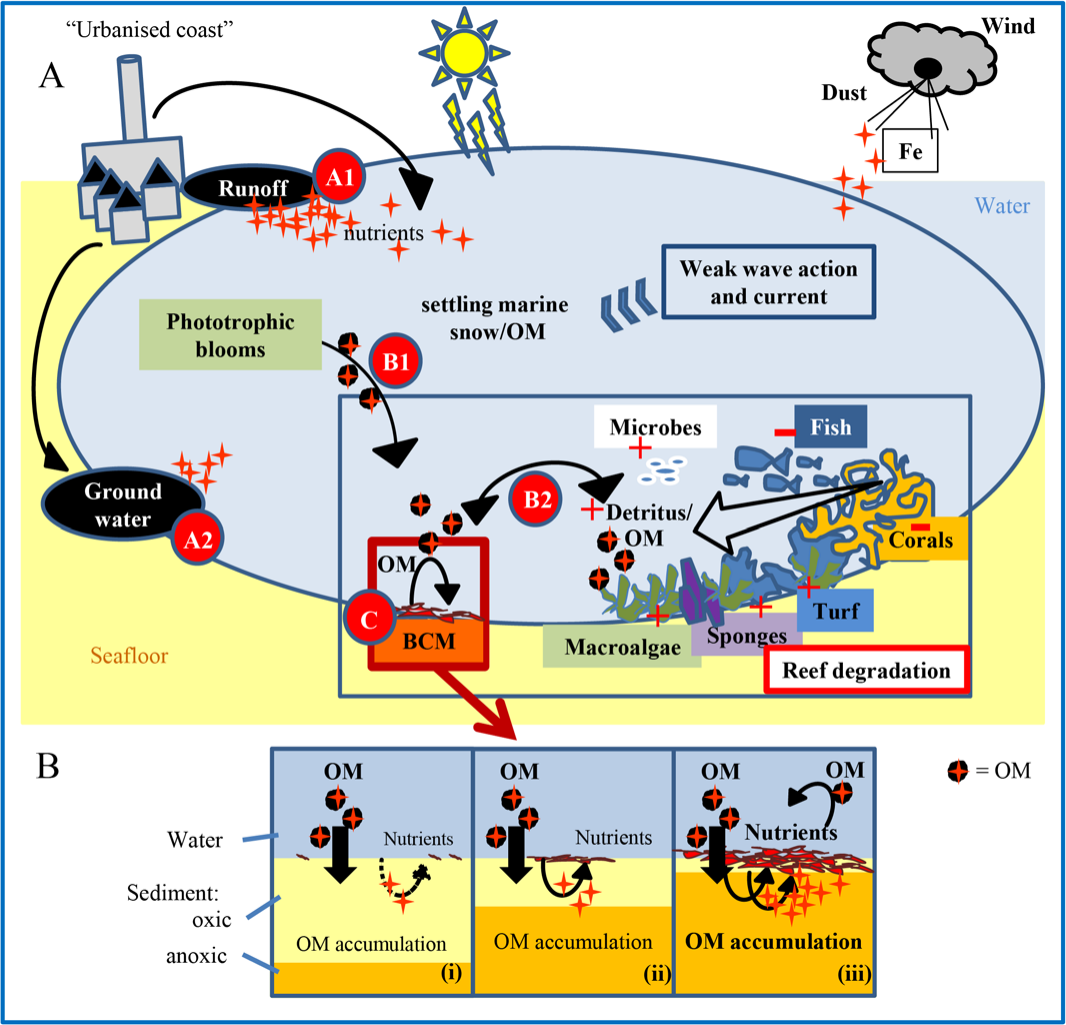
Proposed explanatory model. (A) Proposed model of sources and cycle of nutrients stimulating BCM growth. Nutrient inputs from land runoff (A1) or groundwater seepage (A2) cause benthic and planktonic phototrophic blooms. Fe is largely available as a result of long-term Fe addition by the African dust [54]. The blooms decay and produce particulate OM (B1) which settles on the seafloor as a function of wave action and current. The coral reef community takes up, produces and releases OM (B2) [18,33,50,68]. Reef degradation leads to a detritus-based food web and enhances OM accumulation on the seafloor. Increased OM loading leads to BCM growth via the release of nutrients from the microbial degradation of OM (C). (B) Schematic drawing of the water/sediment interface. Drawings from far away from (i), near to (ii), and middle of (iii) BCM patches are used as analogs for what we anticipate as OM accumulates over time. Increased OM concentration in the sediments results in a thinner oxygenated surface layer and an increased nutrient release, which triggers BCM growth. When BCMs develop, they produce OM and trap OM from the water column to sustain their growth and expand.

Sediments associated with mat-forming and rhizophytic benthic algae on coral reefs have been shown to function as localised nutrient sources, making sustained growth possible despite the oligotrophic water column [18]. Similarly, *L*. *majuscula* growth may be maintained by additional inputs of nutrients through sediment efflux in seagrass beds in Moreton Bay [29]. Given that (i) BCMs were prominent on reefs with more organically rich sediments, and (ii) the mat types growing on sediments developed within days when OM was added to the sediments, our results suggest that the nutrient source for BCMs originates from OM that has settled on the seafloor and is decomposed by microbial degradation as proposed in Fig 6B. Due to the degradation, an anoxic zone develops at the sediment surface. The ensuing Fe^3+^ reduction leads to release of Fe^3+^-bound phosphate to the water column, and possibly also of Fe^2+^ [46]. This local nutrient release from the benthos subsequently stimulates the growth of BCMs. Cyanobacterial mats produce OM via photosynthesis, excretion and cell decomposition. Aerobic heterotrophs and anaerobic sulphate-reducing bacteria mainly respire the produced OM [47]. An indication of these photosynthetic and remineralising processes within the BCMs is the elevated O_2_ concentrations and the anoxia at the mat surface during day and night, respectively. Overtime, OM accumulates under the mat, leading to further BCM growth and patch expansion. To enhance this process, many cyanobacteria excrete extracellular polymeric substances mainly composed of polysaccharides, which form sticky structures capable of catching detritus from the water column [48].

While elevated nutrient concentrations were found above BCMs growing over both hard substratum and sediments, we did not conduct an organic enrichment experiment on BCMs occurring on hard substrates. However, similar mechanisms of BCM growth enhancement by OM may operate here too. In Curaçao, most BCMs covering hard substratum grew over turfs and macroalgae. Turfs and macroalgae can act as sediment traps, accumulate particulate OM on or within their canopies [33,49] and provide dissolved OM by photosynthesis and degradation [50,51]. Schaffelke [33] demonstrated that the layer of particulate matter deposited on the thalli of *Sargassum* was sufficient to supplement their nutrient supply. She postulated that a nutrient-rich diffusive boundary layer was created on the thallus surface by an epiphytic microbial community that remineralised the bound nutrients. Likewise, the OM accumulating on hard substratum could provide a nutrient base for BCMs in this study.

The results of this study also show that BCMs (i) were prominent in sheltered reefs close to the urbanised areas, highlighting the importance of urban and industrial development and wave energy, and (ii) were more abundant in the warm/rainy season than in cold/dry season. Elevated temperature and rainfall are known to favour benthic cyanobacteria, as shown in Moreton Bay [3,29,44]. While high wave energy can physically remove BCMs growing loosely on the seabed [21,52], it could also prevent the deposition and accumulation of OM on the seafloor. Wave-exposed habitats are commonly composed of coarse carbonate sand, with low organic content and low pore-water nutrient concentrations [18]. In such habitats, advection from the water column has been considered as the primary source of nutrients for benthic organisms, while, at protected sites, the contribution of nutrients from benthic sources increases [18]. Schaffelke [33] found that organic content of particulate matter on *Sargassum* was negatively correlated with water flow. Strong currents will create thinner boundary layers, enhance oxidative remineralisation processes within the sediment, rapidly dilute nutrients released from the seabed, and decrease the exposure time to nutrients and consequently BCM growth.

Very few studies have connected BCM abundance to urbanisation on coral reefs. Only in Moreton Bay was land use directly linked to mat abundances [3,29,44]. Together our data suggest that the distribution and abundance of BCMs in oligotrophic coral reef systems depend on the interplay between the input and production of organics and mineral particles to and in the system (which is influenced by coastal urbanisation and reef degradation), the rate of settling on the surface (which is determined by local hydrodynamics), and the subsequent cascade of heterotrophic microbial processes following the settling. We have integrated these processes into the proposed model in Fig 6A. Both runoff and seepage are known to occur in Curaçao. Gast et al. [53] found NO_x_ and PO_4_^3-^ values to reach up to 0.92 μM and 0.29 μM, respectively, at the entrance of the harbour of the capital city Willemstad. NO_3_^-^ concentrations up to 1612 μM have been measured in ground water [37]. Since the Caribbean has been exposed to Fe-rich influxes of African dust for 40,000 years, water column and sediments may act as reservoirs of Fe in the region [54]. In addition, van Sambeek et al. [37] documented higher iron concentration in the groundwater of volcanic formations in Curaçao. In a Fe-loaded system, N and P may be the limiting nutrients controlling benthic and planktonic phototrophic blooms, which, upon decay, convert nutrients into particulate OM. Thus, nutrients may be transported from land to the reef bound in particulate organic matter, while water column concentrations remain low.

The quantity of nutrients and organics in the water column did not differ between sites with high and low BCM abundance. Nevertheless, the high OM concentrations in BCM-free sediments at sites with high BCM abundance are indicative of periods of high OM loads. Water column nutrients, chlorophyll and organics vary greatly depending on wave action, tides, storms and rainfall [55], making it difficult to accurately assess nutrification status by water column measurements alone. The quantity of organics and nutrients in sediments, however, are thought to be a good proxy for long-term nutrient pollution [56]. In coral reefs, the majority of the productivity occurs on the benthos and nutrients are rapidly converted into biomass by benthic algae or macrophytes [57], even before phytoplankton can accumulate. The produced OM subsequently settles, buries and accumulates in sediments, which serve as repository for both water column and benthic production, as well as detrital materials originating from external sources [56,58–60]. Finally, reef degradation is likely to enhance this OM accumulation by forming increasingly detritus-based over grazing-based food webs, with a switch in energy allocation from fish to microbes [61].

Many studies underscore the role of nutrients regenerated from OM degradation in supporting primary producers on coral reefs and other marine coastal systems [60,62–65]. Future studies need to include the origins of the OM, the role of other potentially limiting nutrients (e.g. Fe, molybdenum) and microbial processes. The combined influences of urbanisation, temperature, rainfall, hydrodynamics and reef degradation in mediating BCM abundance suggest that BCMs will be more common under environmental conditions associated with anthropogenic impacts and global climate change. For example, larger and more intense rainfall events associated with climate change mobilize nutrients on land and increase nutrient inputs from land runoff in receiving waters [19]. Management approaches that prevent the input of nutrients generated on land and restore food webs should reduce BCM proliferation on coral reefs. Because OM also kills coral tissue when abundant in dissolved form in the water column [66] or within sediments encroaching corals [67], our results add on to the multiple detrimental effects of increasing OM on coral reefs and suggest that organic loading of sediments and other benthos should be routinely monitored.

## Supporting Information

S1 Datasets Relevant datasets. (XLSX)

S1 Fig Seawater temperatures at the study sites. Seawater temperatures on the reef slope at 10 m depth at the 4 low BCM abundance (left column) and the 4 high BCM abundance (right column) sites from September 2010 to June 2012. Site abbreviations: BY0 = Carmabi buoy 0, PB = Pest Bay, JT = Jan Thiel, SP2 = South Port station 2, MP = Marie Pompon, BO = Boca (Bullen Bay), CM = Cap Malmeeuw, BY3 = Carmabi buoy 3; HB = Holiday Beach; M = Santa Martha Bay; PK = Playa Kalki; Cap Lÿhoek; SW = Spanish Water station 1+2.
(TIF)

S1 Table Statistical output table for BCM abundance scores. PERMANOVA results of the effects of season (fixed), time periods nested within season (random), urbanisation and wave height (covariates) on BCM abundance scores.
(DOC)

S2 Table Statistical output table for clodcard dissolution rates. PERMANOVA results of the effects of season (fixed), BCM site abundance (fixed) and site nested within BCM site abundance (random) on Clodcard dissolution rates.
(DOC)

S3 Table Statistical output table for nutrient concentrations in the water column. PERMANOVA results of the effects of season, BCM site abundance, depth, BCM patch (i.e. above BCM vs. above BCM-free substrate) and site nested within BCM abundance (as appropriate) on NOx and PO_4_^3-^ concentrations in the surface water (SW), open ocean water (OW), intermediate water (IW) and bottom water (BW).
(DOC)

S4 Table Statistical output table for particulate organic matter concentrations in the water column. PERMANOVA results of the effects of BCM site abundance (fixed) and site nested within BCM site abundance (random) on concentration of particulate organic matter in the water column.
(DOC)
