# Supplementary figures and images for: Organic Matter Degradation Drives Benthic Cyanobacterial Mat Abundance on Caribbean Coral Reefs
Source: PLoS One. 2015 May 5;10(5):e0125445. doi: 10.1371/journal.pone.0125445 (PMC4420485; doi:10.1371/journal.pone.0125445)

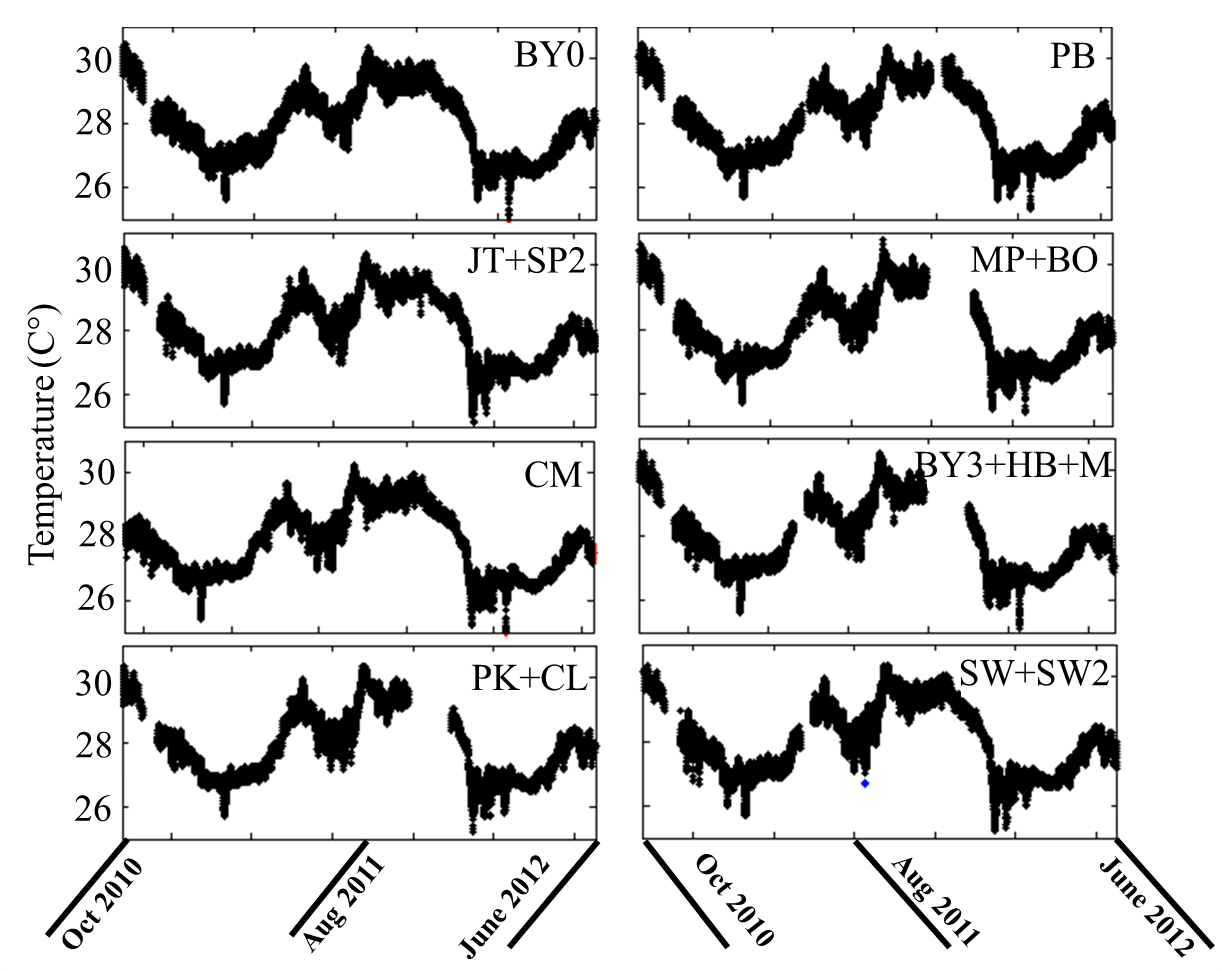

Supplement: S1 Fig — Seawater temperatures on the reef slope at 10 m depth at the 4 low BCM abundance (left column) and the 4 high BCM abundance (right column) sites from September 2010 to June 2012. Site abbreviations: BY0 = Carmabi buoy 0, PB = Pest Bay, JT = Jan Thiel, SP2 = South Port station 2, MP = Marie Pompon, BO = Boca (Bullen Bay), CM = Cap Malmeeuw, BY3 = Carmabi buoy 3; HB = Holiday Beach; M = Santa Martha Bay; PK = Playa Kalki; Cap Lÿhoek; SW = Spanish Water station 1+2. (TIF) [file pone.0125445.s002.tif]
