# Supplementary material for: Organic Matter Degradation Drives Benthic Cyanobacterial Mat Abundance on Caribbean Coral Reefs
Source: PLoS One. 2015 May 5;10(5):e0125445. doi: 10.1371/journal.pone.0125445 (PMC4420485; doi:10.1371/journal.pone.0125445)
Supplement: S1 Table — PERMANOVA results of the effects of season (fixed), time periods nested within season (random), urbanisation and wave height (covariates) on BCM abundance scores. (DOC) [file pone.0125445.s003.doc]

**S1 Table. Statistical output table for BCM abundance scores.**

|  |  |  |  |
| --- | --- | --- | --- |
| **Source in relation to cyanobacterial abundance** | **df** | **Pseudo-F** | **P(perm)** |
|  |  |  |  |
| season (se) | 1 | 3,06 | 0,001*** |
| time points (nested in season) (ti(se)) | 2 | 1,04 | 0,374 |
| urbanisation status (ur) | 1 | 53,98 | 0,001*** |
| wave heights (wa) | 1 | 102,57 | 0,001*** |
| ur x wa | 1 | 3,99 | 0,037* |
| ur x se | 1 | 0,03 | 0,844 |
| wa x se | 1 | 0,10 | 0,763 |
| ur x ti(se) | 2 | 1,05 | 0,357 |
| wa x ti(se) | 2 | 2,14 | 0,12 |
| ur x wa x se | 1 | 3,02 | 0,099 |
| ur x wa x ti(se) | 2 | 0,58 | 0,536 |
|  |  |  |  |

PERMANOVA results of the effects of season (fixed), time periods nested within season (random), urbanisation and wave height (covariates) on BCM abundance scores.
