# Supplementary material for: Organic Matter Degradation Drives Benthic Cyanobacterial Mat Abundance on Caribbean Coral Reefs
Source: PLoS One. 2015 May 5;10(5):e0125445. doi: 10.1371/journal.pone.0125445 (PMC4420485; doi:10.1371/journal.pone.0125445)
Supplement: S2 Table — PERMANOVA results of the effects of season (fixed), BCM site abundance (fixed) and site nested within BCM site abundance (random) on Clodcard dissolution rates. (DOC) [file pone.0125445.s004.doc]

**S2 Table. Statistical output table for clodcard dissolution rates.**

|  |  |  |  |
| --- | --- | --- | --- |
| **Source in relation to clodcard dissolution rates** | **df** | **Pseudo-F** | **P(perm)** |
|  |  |  |  |
| season (se) | 1 | 4,06 | 0,091 |
| BCM abundance (BCM) | 1 | 104,95 | 0,026* |
| site nested in BCM (si(BCM)) | 6 | 9,75 | 0,001*** |
| BCM x se | 1 | 0,02 | 0,89 |
| se x si(BCM) | 6 | 18,13 | 0,001*** |
|  |  |  |  |

PERMANOVA results of the effects of season (fixed), BCM site abundance (fixed) and site nested within BCM site abundance (random) on Clodcard dissolution rates.
