# Supplementary material for: Organic Matter Degradation Drives Benthic Cyanobacterial Mat Abundance on Caribbean Coral Reefs
Source: PLoS One. 2015 May 5;10(5):e0125445. doi: 10.1371/journal.pone.0125445 (PMC4420485; doi:10.1371/journal.pone.0125445)
Supplement: S3 Table — PERMANOVA results of the effects of season, BCM site abundance, depth, BCM patch (i.e. above BCM vs. above BCM-free substrate) and site nested within BCM abundance (as appropriate) on NOx and PO4 3- concentrations in the surface water (SW), open ocean water (OW), intermediate water (IW) and bottom water (BW). (DOC) [file pone.0125445.s005.doc]

**S3 Table. Statistical output table for nutrient concentrations in the water column.**

|  |  |  |  |  |  |  |  |
| --- | --- | --- | --- | --- | --- | --- | --- |
|  |  | **NOx** | | | **PO_4_^3-^** | | |
|  | **Source in relation to nutrient concentrations** | **df** | **Pseudo-F^a^** | **P(perm)** | **df** | **Pseudo-F^a^** | **P(perm)** |
|  |  |  |  |  |  |  |  |
| **SW** | season (se) | 1 | 0,00 | 0,987 | 1 | 0,85 | 0,437 |
|  | BCM abundance (BC) | 1 | 2,68 | 0,161 | 1 | 1,11 | 0,258 |
|  | site nested in BCM (si(BC)) | 6 | 0,24 | 0,962 | 6 | 0,88 | 0,615 |
|  | se x BC | 1 | 2,17 | 0,188 | 1 | 0,86 | 0,448 |
|  | se x si(BC) | 6 | 1,06 | 0,396 | 6 | 0,97 | 0,518 |
|  |  |  |  |  |  |  |  |
| **OW** | season (se) | 1 | 0,18 | 0,687 | 1 | 1,54 | 0,294 |
|  | BCM abundance (BC) | 1 | 0,05 | 0,81 | 1 | 0,32 | 0,686 |
|  | site nested in BCM (si(BC)) | 6 | 0,41 | 0,873 | 6 | 1,42 | 0,233 |
|  | se x BC | 1 | 2,02 | 0,21 | 1 | 0,30 | 0,667 |
|  | se x si(BC) | 6 | 0,34 | 0,899 | 6 | 1,84 | 0,121 |
|  |  |  |  |  |  |  |  |
| **IW** | season (se) | 1 | 0,02 | 0,882 | 1 | 2,36 | 0,179 |
|  | BCM abundance (BC) | 1 | 4,35 | 0,071 | 1 | 0,68 | 0,553 |
|  | Site nested in BCM (si(BC)) | 6 | 1,05 | 0,424 | 6 | 2,15 | 0,051 |
|  | depth (de) | 1 | 0,23 | 0,651 | 1 | 2,77 | 0,128 |
|  | se x BC | 1 | 0,09 | 0,776 | 1 | 0,95 | 0,405 |
|  | se x de | 1 | 0,37 | 0,548 | 1 | 2,25 | 0,182 |
|  | BC x de | 1 | 7,60 | 0,04* | 1 | 0,08 | 0,769 |
|  | se x si(BC) | 6 | 2,67 | 0,028* | 6 | 2,02 | 0,055 |
|  | si(BC) x de | 6 | 0,39 | 0,895 | 6 | 0,16 | 0,991 |
|  | se x BC x de | 1 | 0,03 | 0,886 | 1 | 0,08 | 0,774 |
|  | se x si(BC) x de | 6 | 1,67 | 0,15 | 6 | 0,12 | 0,997 |
|  |  |  |  |  |  |  |  |
| **BW** | season (se) | 1 | 6,96 | 0,041* | 1 | 6,88 | 0,05 |
|  | BCM abundance (BC) | 1 | 8,80 | 0,005** | 1 | 0,20 | 0,632 |
|  | Site nested in BCM (si(BC)) | 6 | 1,18 | 0,308 | 6 | 3,34 | 0,004** |
|  | depth (de)^b^ | 1 | 6,22 | 0,047* | 1 | 4,34 | 0,068 |
|  | patch (pa) | 1 | 11,26 | 0,011* | 1 | 13,76 | 0,01** |
|  | se x BC | 1 | 8,16 | 0,043* | 1 | 0,21 | 0,634 |
|  | se x de^b^ | 1 | 1,19 | 0,32 | 1 | 1,74 | 0,219 |
|  | se x pa | 1 | 2,98 | 0,137 | 1 | 8,10 | 0,029* |
|  | BC x de^b^ | 1 | 7,87 | 0,032* | 1 | 0,04 | 0,837 |
|  | BC x pa | 1 | 0,15 | 0,701 | 1 | 0,00 | 0,991 |
|  | de^b^ x pa | 1 | 4,25 | 0,102 | 1 | 3,40 | 0,113 |
|  | se x si(BC) | 6 | 0,71 | 0,624 | 6 | 2,91 | 0,009** |
|  | si(BC) x de^b^ | 6 | 0,40 | 0,886 | 6 | 1,73 | 0,102 |
|  | si(BC) x pa | 6 | 1,18 | 0,331 | 6 | 1,81 | 0,098 |
|  | se x BC x de^b^ | 1 | 1,34 | 0,315 | 1 | 0,02 | 0,911 |
|  | se x BC x pa | 1 | 1,71 | 0,241 | 1 | 0,02 | 0,863 |
|  | se x de^b^ x pa | 1 | 0,62 | 0,467 | 1 | 0,65 | 0,463 |
|  | BC x de^b^ x pa | 1 | 8,76 | 0,03* | 1 | 0,06 | 0,827 |
|  | se x si(BC) x de^b^ | 6 | 0,22 | 0,975 | 6 | 1,66 | 0,112 |
|  | se x si(BC) x pa | 6 | 1,04 | 0,413 | 6 | 1,53 | 0,159 |
|  | si(BC) x de^b^ x pa | 6 | 0,26 | 0,957 | 6 | 1,11 | 0,363 |
|  | se x BC x de^b^ x pa | 1 | 0,34 | 0,602 | 1 | 0,26 | 0,647 |
|  | se x si(BC) x de^b^ x pa | 6 | 0,48 | 0,825 | 6 | 1,25 | 0,287 |
|  |  |  |  |  |  |  |  |

^a^ * = *P* < 0.05, ** *P* < 0.01, *** *P* < 0.001; ^b^ The effect of depth is confounded with the effects of substratum and mat types since brown-colored mats over sand and red-colored mat over hard substrates were sampled at 5 and 15 m depths, respectively.

PERMANOVA results of the effects of season, BCM site abundance, depth, BCM patch (i.e. above BCM vs. above BCM-free substrate) and site nested within BCM abundance (as appropriate) on NOx and PO_4_^3-^ concentrations in the surface water (SW), open ocean water (OW), intermediate water (IW) and bottom water (BW).
