# Supplementary material for: Organic Matter Degradation Drives Benthic Cyanobacterial Mat Abundance on Caribbean Coral Reefs
Source: PLoS One. 2015 May 5;10(5):e0125445. doi: 10.1371/journal.pone.0125445 (PMC4420485; doi:10.1371/journal.pone.0125445)
Supplement: S4 Table — PERMANOVA results of the effects of BCM site abundance (fixed) and site nested within BCM site abundance (random) on concentration of particulate organic matter in the water column. (DOC) [file pone.0125445.s006.doc]

**S4 Table. Statistical output table for particulate organic matter concentrations in the water column.**

|  |  |  |  |
| --- | --- | --- | --- |
| **Source in relation to of particulate organic matter** | **df** | **Pseudo-F** | **P(perm)** |
|  |  |  |  |
| BCM abundance (BCM) | 1 | 1,56 | 0,245 |
| site nested in BCM (si(BCM)) | 6 | 2,19 | 0,074 |
|  |  |  |  |

PERMANOVA results of the effects of BCM site abundance (fixed) and site nested within BCM site abundance (random) on concentration of particulate organic matter in the water column.
